# Continuous and bilevel positive airway pressure may improve radiotherapy delivery in patients with intra-thoracic tumors

**DOI:** 10.1016/j.ctro.2024.100784

**Published:** 2024-04-20

**Authors:** J. Elshof, C.M. Steenstra, A.G.H. Niezink, P.J. Wijkstra, R. Wijsman, M.L. Duiverman

**Affiliations:** a Department of Pulmonary Diseases/Home Mechanical Ventilation, University of Groningen, University Medical Center Groningen, Groningen, The Netherlands; b Groningen Research Institute for Asthma and COPD, University of Groningen, Groningen, The Netherlands; c Department of Radiation Oncology, University of Groningen, University Medical Center Groningen, Groningen, The Netherlands

**Keywords:** BiPAP, CPAP, Non-invasive ventilation, Radiotherapy

## Abstract

**Background:**

Minimizing tumor motion in radiotherapy for intra-thoracic tumors reduces side-effects by limiting radiation exposure to healthy tissue. Continuous or Bilevel Positive Airway Pressure (CPAP/BiPAP) could achieve this, since it could increase lung inflation and decrease tidal volume variability. We aim to identify the better CPAP/BiPAP setting for minimizing tumor motion.

**Methods:**

In 10 patients (5 with lung cancer, 5 with other intra-thoracic tumors), CPAP/BiPAP was tested with the following settings for 10 min each: CPAP 5, 10 and 15 cmH_2_O and BiPAP 14/10 cmH_2_O with a lower (7 breaths/min) and higher back-up respiratory rate (BURR initially 1 breath/min above the spontaneous breathing frequency, with the option to adjust if the patient continued to initiate breaths). Electrical impedance tomography was used to analyse end-expiratory lung impedance (EELI) as an estimate of end-expiratory lung volume and tidal impedance variation (TIV) as an estimate of tidal volume.

**Results:**

Nine out of ten patients tolerated all settings; one patient could not sustain CPAP-15. A significant difference in EELI was observed between settings (χ^2^ 22.960, p < 0.001), with most increase during CPAP-15 (median (IQR) 1.03 (1.00 – 1.06), normalized to the EELI during spontaneous breathing). No significant differences in TIV and breathing variability were found between settings.

**Conclusions:**

This study shows that the application of different settings of CPAP/BiPAP in patients with intra-thoracic tumors is feasible and tolerable. BiPAP with a higher BURR may offer the greatest potential for mitigating tumor motion among the applied settings, although further research investigating tumor motion should be conducted.

## Highlights

• Application of CPAP and BiPAP in patients with intra-thoracic tumors is feasible and tolerable.
• Both CPAP and BiPAP may increase end-expiratory lung volume.
• Notably, BiPAP with a frequency 3.5 breath/min above spontaneous breathing shows promise, as it may reduce tidal volumes.

## Introduction

Radiotherapy plays a crucial role in lung cancer treatment, with approximately three-quarters of lung cancer patients requiring radiotherapy at some point during the course of their disease [1]. Despite recent advancements in radiotherapy techniques that have improved the accuracy of radiation delivery, breathing-induced tumor motion remains a challenge: motion of intra-thoracic tumors may exceed 3 cm [2], [3], [4]. These tumor motion amplitudes are taken into account in radiotherapy treatment planning by increasing treatment volume (i.e. creating an internal target volume) to ensure proper irradiation of the tumor during the entire breathing cycle. In turn, this enlargement of the irradiated volume may lead to additional radiation exposure for healthy tissues, increasing the likelihood of radiation-induced side effects, such as radiation pneumonitis and acute esophageal toxicity [5], [6], [7]. Several types of management strategies have been developed to mitigate the effects of breathing-induced motion, such as respiratory gating, breath-hold techniques or patient coaching [8]. However, these methods highly rely on patient compliance and may therefore be challenging to perform, particularly for patients with underlying lung disease.

Therefore, other methods to mitigate tumor motion are worth exploring such as the application of Continuous Positive Airway Pressure (CPAP) via a mask. CPAP might reduce tumor motion by applying a constant positive airway pressure, resulting in increased lung volumes, more diaphragm flattening and consequently reduced diaphragm motion [9]. In addition, this increased lung inflation displaces healthy lung tissue further away from the tumor and radiation beam and decrease lung density, thereby reducing incidental radiation dose to healthy tissues [10]. Earlier studies examining the application of CPAP at a pressure level of 15 cmH_2_O in patients receiving radiation therapy found a decrease in tumor movement and a decrease in both lung and heart radiation dose compared to spontaneous breathing [10], [11]. A disadvantage of CPAP is the unpredictable breathing pattern, as the patients determines tidal volume and breathing frequency. In contrast, with the application of Bilevel Positive Airway Pressure (BiPAP) via a mask enables regulation of the delivered tidal volume. This regulation is established by the difference between a preset higher pressure level during inspiration (IPAP) and a preset lower pressure level during expiration (EPAP). Furthermore, with BiPAP, a back-up respiratory rate (BURR) can be set. If a patient’s breathing frequency exceeds the BURR, the patient self-regulates their respiratory rate; conversely, when the opposite occurs, BiPAP facilitates control over the patient’s respiratory pattern. Next to the effects on lung inflation, BiPAP could thereby reduce the variability of the patient’s breathing pattern, which may result in lower radiation doses for organs at risk. However, patients might experience more difficulty in adapting to BiPAP compared to CPAP.

While no previous studies have investigated the impact of BiPAP on tumor motion in patients undergoing radiotherapy, a number of pilot studies demonstrated that BiPAP is feasible in both healthy subjects and patients with intra-thoracic cancer using varying breathing frequencies ranging from 16 to 30 breaths per minute. The application of BiPAP resulted in reduced breathing variability compared to spontaneous breathing [12], [13], [14], [15], [16]. However, notable differences were observed between studies related to the effects of BiPAP on diaphragm or tumor motion [14], [15], [16], [17]. No research has compared different settings of both CPAP and BiPAP in patients undergoing radiotherapy.

Therefore, the aim of this pilot study is to investigate the feasibility of short-term use of some different settings of CPAP and BiPAP and their impact on estimates of end-expiratory lung volume, tidal volume and breathing pattern variability in patients undergoing radiotherapy for intra-thoracic tumors (paired design). Our objective is to identify the better CPAP or BiPAP setting among the applied configurations, aiming to guide further research to ultimately minimize tumor motion and reduce radiation exposure to healthy tissues.

## Methods

### Study design

This prospective observational study was performed in the University Medical Center Groningen from June 2021 to December 2022. Patients had to meet the following criteria to be included: ≥18 years of age, having stage III or IV (non–)small cell lung cancer, esophageal cancer or malignant lymphoma currently undergoing radiotherapy with curative intent, and a WHO performance status between 0 and 2. Exclusion criteria included facial deformations that prevented the right mask fit, severe heart failure (left ventricular ejection fraction < 30 %) or planned for radiotherapy with a fraction dose higher than 3 Gray.

The study protocol was reviewed and approved by the medical ethics committee of the University Medical Center Groningen. Written informed consent was obtained from all study participants.

### Study procedures

Study participants were invited to the outpatient clinic once. Patients were fitted with appropriately sized nose mask and were instructed to ensure their mouths remained closed during CPAP/BiPAP application to prevent leakage. A short protocol (±20 min) was followed to acclimatise the participant with CPAP and BiPAP on the ventilator (BiPAP A30, Philips Respironics, Murrysville, PA).

After the acclimatisation protocol, the following settings were administered with room air for 10 min each, with 5 min spontaneous breathing in between: baseline (spontaneous breathing without CPAP/BiPAP), CPAP with a level of 5, 10 and 15 cmH_2_O (CPAP-5, CPAP-10 and CPAP-15, respectively) and BiPAP with an inspiratory pressure of 14 cmH_2_O and expiratory pressure of 10 cmH_2_O with a lower (7 breaths/min) and higher (1 breath/min above the spontaneous breathing frequency) back-up respiratory rate (BURR). The spontaneous breathing frequency was determined while administering BiPAP with the lower BURR. Initially, the BURR during BiPAP with a higher BURR was set at 1 breath per minute above this established spontaneous breathing frequency, with the option for adjustment in case the patient continued to initiate breaths.

### Data collection

Electrical impedance tomography (EIT) using the Swisstom BB2 device (Swisstom, Landquart, Switzerland) was recorded to estimate lung volumes. Gas exchange (transcutaneous partial pressure of carbon dioxide (PtcCO_2_) and oxygen saturation (SaO_2_)) and heart rate were monitored (SenTec AG, Therwil, Switzerland) for safety reasons. The lower limit for the PtCO_2_ was cautiously set at 3.0 kPa. The number of patient triggered breaths during BiPAP with a high BURR were read out from the ventilator using dedicated software (DirectView, Philips Respironics). The CPAP or BiPAP setting was considered tolerable if the patient could sustain it for 10 min. Each setting was rated by the participant on a visual analogue scale ranging from 0 (comfortable) to 10 (uncomfortable).

### Analyses and statistics

The EIT data was imported into Matlab (v2018a, The MathWorks Inc., Natick, MA, USA) using Ibex software (v1.6, Swisstom, Landquart, Switzerland). The global impedance signal was used for further analysis. Initially, the signal was examined visually and any disturbances caused by movement were eliminated. Subsequently, a low-pass filter with a cut-off frequency of 1 Hz was applied to eliminate the impact of cardiac activity while retaining the ventilatory component of the signal.

Tidal impedance variation (TIV), defined as the impedance change during a tidal breath, was used as to estimate tidal volume [18], [19]. The end-expiratory lung impedance (EELI) was used as to estimate end-expiratory lung volume [19], [20], [21]. For each patient, the TIV and EELI at each CPAP/BiPAP setting were compared with their corresponding measurements obtained during a baseline measurement while free breathing (thus without CPAP or BiPAP). This ensure consistent comparison between patients since absolute impedance values are irrelevant [22]. The variability of the patient’s breathing pattern was evaluated for both estimated tidal volume and frequency. Tidal volume variability was determined by dividing the interquartile range of all TIV values at that particular setting by the mean TIV at baseline, ensuring comparability between subjects and across various CPAP/BiPAP settings. Similarly, variability of the breathing frequency was calculated by dividing the interquartile range of respiratory rates at that specific setting by the mean respiratory rate at baseline.

All parameters were averaged over the final 5 min of each specific setting. The Friedman test was used to compare all CPAP/BiPAP settings. A p-value < 0.05 was considered as statistically significant. In cases where the Friedman test was significant, Dunn’s pairwise post hoc tests with Bonferroni adjustments were conducted to determine which settings displayed significant differences. Mann-Whitney tests were performed to compare differences between patients diagnosed with lung cancer and those with other intra-thoracic tumors. Spearman’s correlation test was used to determine the relationship between the number of triggered breaths and breathing variability.

## Results

Ten patients were included in the study: five with lung cancer, one with oesophageal cancer and four with malignant lymphoma (Table 1).

**Table 1:** Characteristics of study participants.

#	Sex	Age (years)	WHO score	Diagnosis		Relevant comorbidities
				Type of tumor	Stage	
1	M	67	1	NSCLC	cT4N1M0	COPD GOLD 3, hypertension
2	M	33	1	Hodgkin lymphoma	II	Obesity, hypertension
3	M	56	1	NSCLC	cT1cN2M1b	COPD GOLD 4, diabetes mellitus, hypertension
4	M	61	2	NSCLC	cT4N2M1b	OSAS
5	M	70	1	NSCLC	cT2aN2M0	diabetes mellitus, cholecystectomy
6	M	80	1	NSCLC	cT2aN3M0	COPD GOLD 3, essential thrombocythemia
7	M	58	0	Esophageal carcinoma	cT3N1M0	None
8	M	34	0	Hodgkin lymphoma	IIB	None
9	F	23	1	Hodgkin lymphoma	IIB	None
10	M	20	1	Hodgkin lymphoma	IIA	None

***Abbreviations****: WHO, world health organization performance score; M, male; F, female; NSCLC, non-small-cell lung cancer; COPD, chronic obstructive pulmonary disease; OSAS, obstructive sleep apnea syndrome.*

The spontaneous breathing frequency during BiPAP with a lower BURR ranged between 9 and 16 breaths per minute with a median of 12.5. The applied BURR at the BiPAP setting with a higher BURR ranged between 12 and 20 breaths per minute with a median of 16. Consequently, the BURR was set at a median of 3.5 breaths per minute (range 1 – 7) higher than the spontaneous breathing frequency. The quality of the EIT signal during the baseline measurement without CPAP/BiPAP of patient 4 was not sufficient for analysis. Consequently, for this particular patient, all measurements derived from the EIT signal were compared to the mean value of all CPAP/BiPAP settings, rather than using the baseline measurement as a reference.

### Feasibility, comfort and safety

**Fig. 1:**
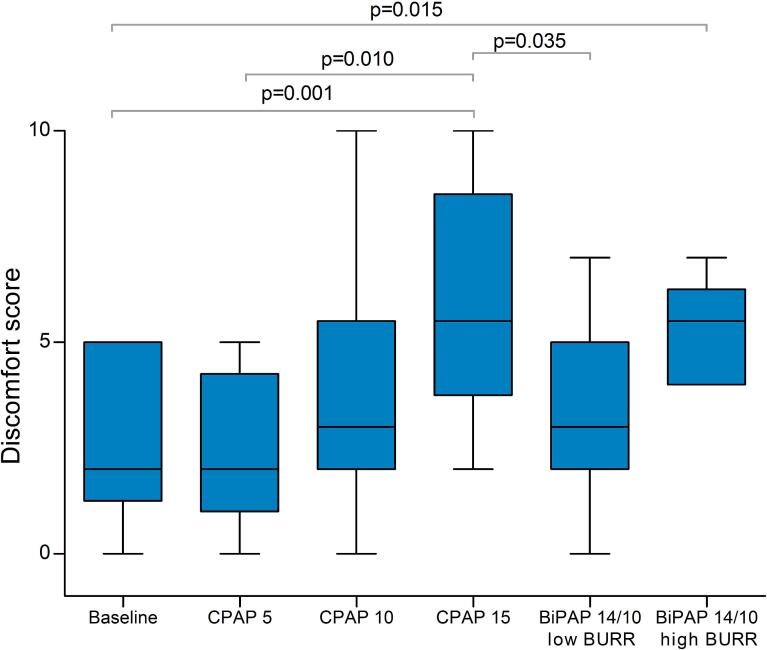
Comfort scores for each setting ranging from 0 (comfortable) to 10 (uncomfortable). Significant differences on the post-hoc analysis are indicated with their respective p-value. The box plots represent the interquartile range with the median, and the whiskers represent the range.

Both CPAP and BiPAP were generally well-tolerated. Nine patients were able to tolerate all settings. One patient did not tolerate CPAP-15, while tolerating the other settings. There was a significant difference in comfort scores between settings (Fig. 1, χ^2^ 28.502, p < 0.001). CPAP-15 was rated as the most uncomfortable setting in six out of ten patients.

Due to safety precautions and our intention to be most cautious, the measurements were discontinued after seven minutes in one patient during BiPAP with a high BURR. This decision was made because the patient’s PtCO_2_ level dropped to 2.9 kPa. Importantly, the patient did not experience any symptoms of hyperventilation, such as dizziness, tingling and numbness.

There were significant but not clinically relevant differences in PtcCO_2_ and SaO_2_ levels between settings (respectively χ^2^ 25.824, p < 0.001 and χ^2^ 26.894, p < 0.001), while no differences in heart rate was found between settings. Post-hoc analyses revealed that with BiPAP, median (IQR) PtcCO_2_ levels significantly decreased (4.7 (4. 6–––5.0) kPa during CPAP-5 vs. BiPAP lower BURR 4.3 (4.1 – 4.6) kPa, p < 0.001; CPAP-5 vs. BiPAP higher BURR 4.2 (3.9 – 4.7) kPa, p < 0.001) and SaO_2_ levels increased (baseline 96 (94 – 96)% vs. BiPAP lower BURR 98 (97 – 98) %, p = 0.005; baseline vs. BiPAP higher BURR 97 (97 – 99) %, p = 0.002).

### Estimated end-expiratory lung volume

**Fig. 2:**
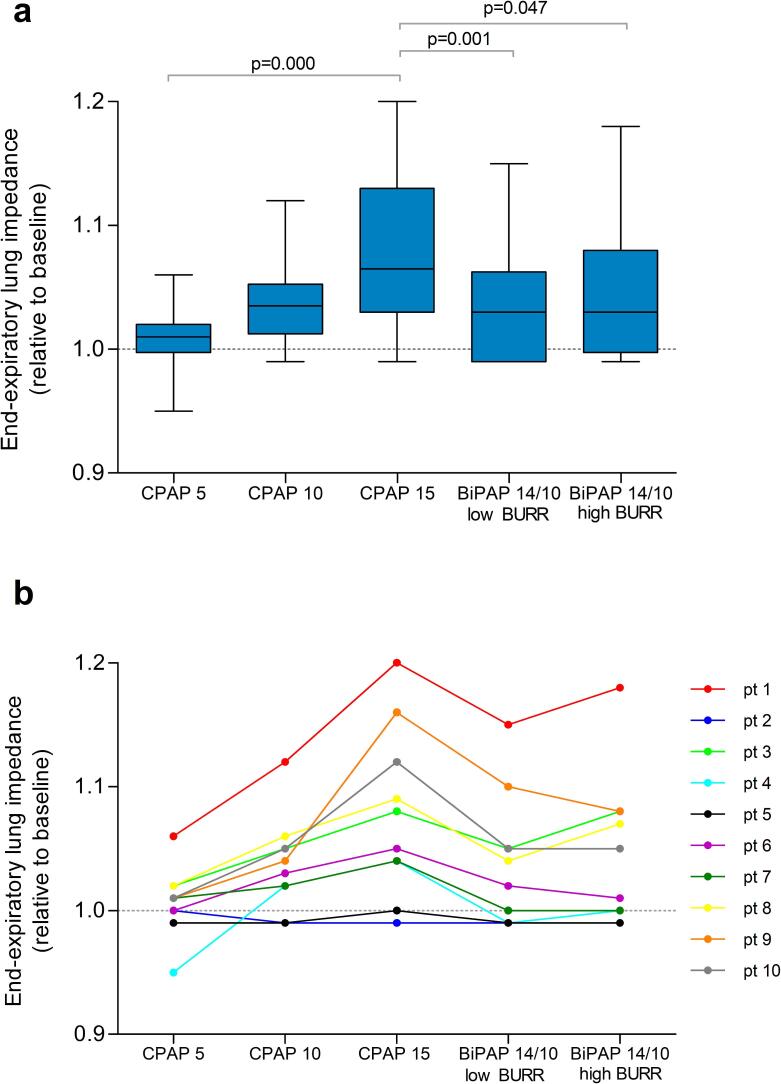
End-expiratory lung impedance as an estimate of end-expiratory lung volume, relative to baseline (indicated by the dotted line) a) box plots represents the interquartile range with the median, and the whiskers represent the range for each setting b) individual levels of end-expiratory lung impedance per setting. This figures shows whether a specific CPAP/BiPAP setting decreases (<1) or increases (>1) estimated end-expiratory lung volume in comparison with baseline and enables comparison of the estimated end-expiratory lung volume between CPAP/BiPAP setting. Significant differences on the post-hoc analysis are indicated with their respective p-value.

A significant difference in EELI was observed between settings (χ^2^ 22.960, p < 0.001). EELI increased most during CPAP-15 (Fig. 2a). The individual levels of EELI per setting can be found in Fig. 2b, revealing a progressive increase in EELI with higher EPAP levels.

### Estimated tidal volume

**Fig. 3:**
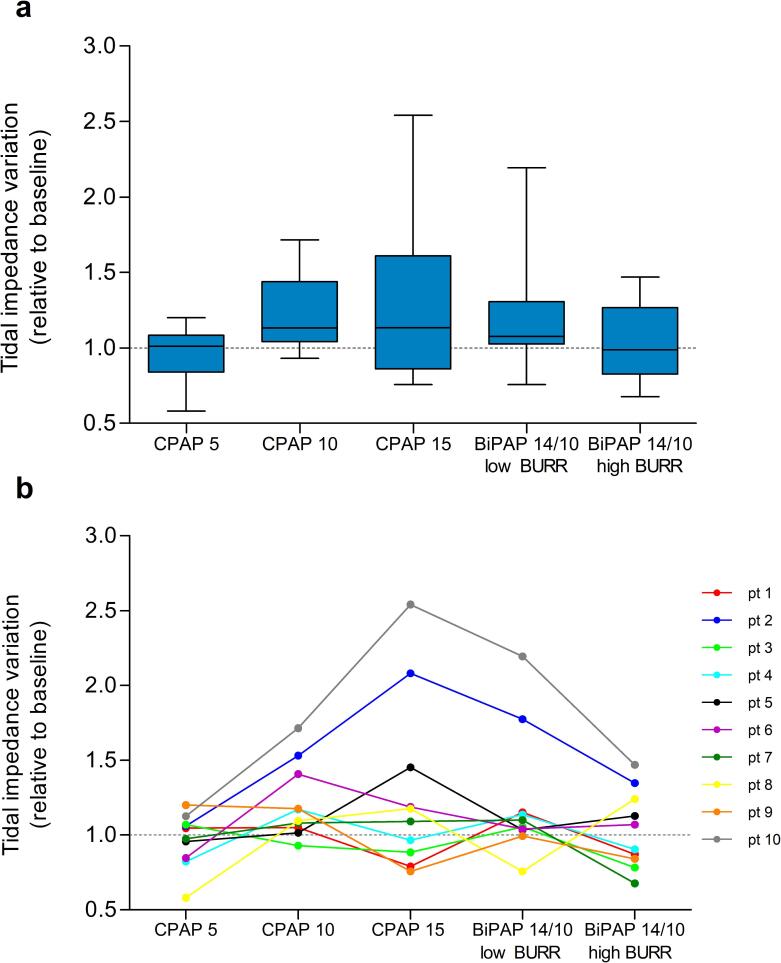
Tidal impedance variation as an estimate of tidal volume, relative to baseline (indicated by the dotted line) a) box plots represents the interquartile range with the median, and the whiskers represent the range for each setting b) individual levels of tidal impedance variation per setting. This figure shows whether a specific CPAP/BiPAP settings decreases (<1) or increases (>1) estimated tidal volume in comparison with baseline and enables comparison of estimated tidal volume between different CPAP/BiPAP settings.

No significant differences in TIV were found between settings (Fig. 3a). Fig. 3b shows the individual TIV per setting compared to their baseline TIV, showing that, although no significant differences were found in TIV values between settings, 50 % of the patients had lower TIV compared to baseline during CPAP-5 and BiPAP with a high BURR.

### Variability in breathing pattern

**Fig. 4:**
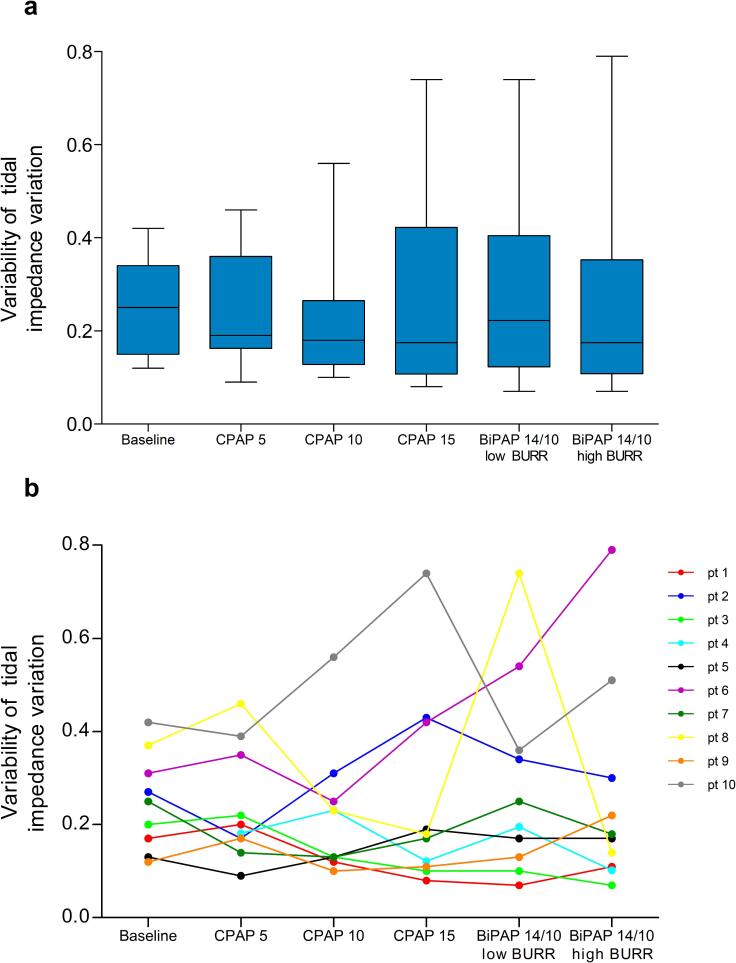
Variability of tidal impedance variation as an estimate of tidal volume variability a) box plots represents the interquartile range with the median, and the whiskers represent the range for each setting b) individual levels of variability of tidal impedance variation per setting.

**Fig. 5:**
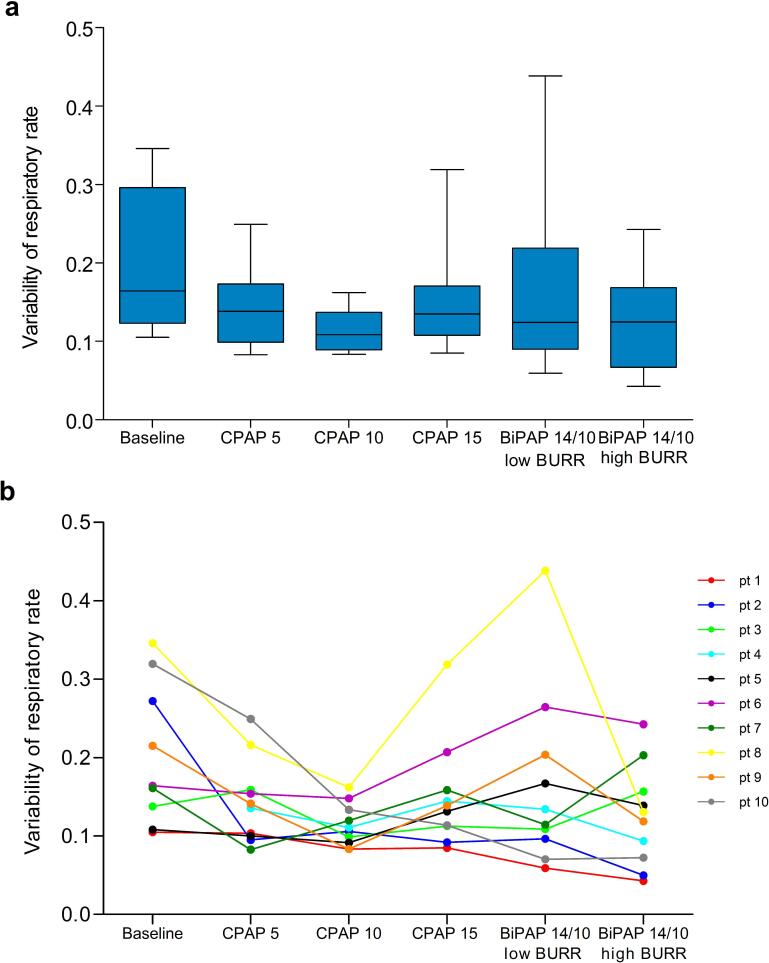
Variability of respiratory rate as a measure of breathing frequency variability a) box plots represents the interquartile range with the median, and the whiskers represent the range for each setting b) individual levels of variability of respiratory rate per setting.

No significant differences in variability were observed for both estimated tidal volume and frequency between settings (Fig. 4a and Fig. 5a, respectively). Individual variability in both estimated tidal volume and frequency can be seen in Fig. 4b and 5b, revealing considerable variation between patients and settings.

**Fig. 6:**
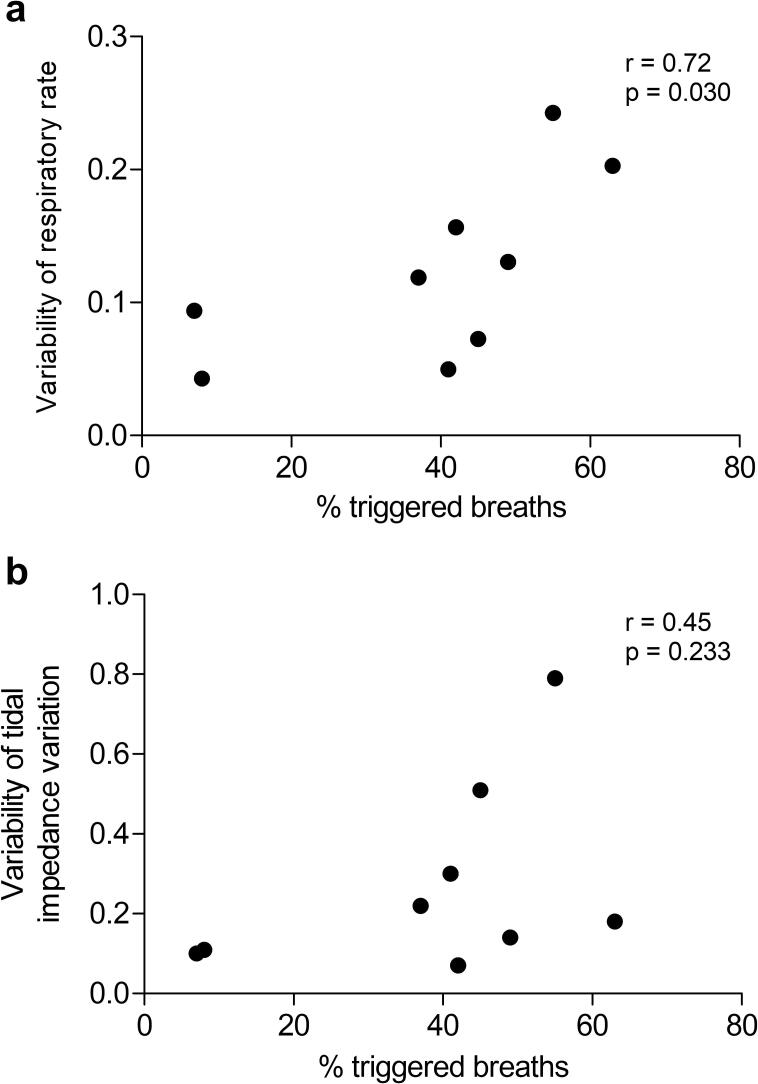
Relationship between the percentage of triggered breaths during BiPAP with a high back-up and a) the respiratory rate as a measure of breathing frequency variability b) the variability of the tidal impedance variation as an estimate of tidal volume variability. Note that there are only nine measurements available, as the percentage of triggered breaths was unavailable for one patient.

The median percentage of triggered breaths during BiPAP with a high BURR was high (42 % (IQR 8 – 51 %)). A significant correlation between the percentage of triggered breaths and the variability of respiratory rate is found during the application of BiPAP with a high BURR (Fig. 6). Although no significant correlation is found between the percentage of triggered breaths and the variability of the tidal impedance variation, Fig. 6b indicates that lower percentages of triggered breaths (<20 %) may be associated with reduced variability in estimated tidal volume.

### Comparison between patients with lung cancer and other intra-thoracic cancers

**Fig. 7:**
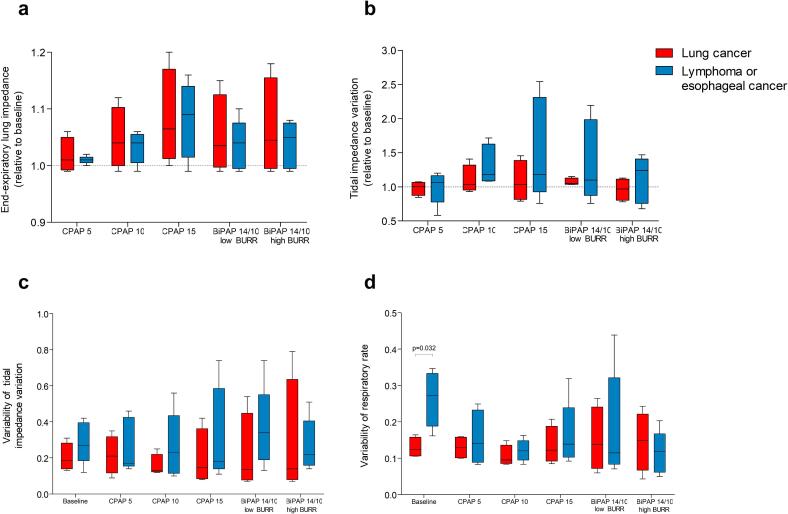
Comparison between patients with lung cancer and other intra-thoracic tumors, box plots represents the interquartile range with the median, and the whiskers represent the range for each setting a) end-expiratory lung impedance as a an estimate of end-expiratory lung volume, relative to baseline (indicated by the dotted line) b) tidal impedance variation as an estimate of tidal volume, relative to baseline (indicated by the dotted line) c) variability of tidal impedance variation as an estimate of tidal volume variability d) variability of respiratory rate as a measure of breathing frequency variability. Significant difference on the Mann-Whitney *U* test is indicated with the p value. Remark: the results of patient 4 were not included in this figure due to missing data at baseline.

Fig. 7 shows the results of the different setting of CPAP/BiPAP on EELI, TIV and variability in breathing pattern for patients with lymphoma or esophageal cancer and those with lung cancer. No significant differences between these patient groups were found, except for a higher variability in respiratory rate at baseline in patients with lymphoma or esophageal cancer compared to those with lung cancer. The median values of EELI, TIV and variability of TIV of seem to be lower in the patients with lung cancer compared to the patients with lymphoma or esophageal cancer in all settings.

## Discussion

This is the first study that evaluated the application of different settings of CPAP and BiPAP in patients with intra-thoracic tumors, aiming to investigate whether the application of positive airway pressure could stabilize breathing and reduce lung motion. We show that the application of CPAP and BiPAP is feasible and tolerable in this patient group. It was observed that increasing levels of EPAP led to increased estimated end-expiratory lung volumes. Despite this increase, we could not detect any significant change in estimated tidal volume and breathing pattern variability with our indirect measurement techniques.

We confirm previous research [10], [11], [23], [24], [25] that has demonstrated that the application of CPAP in patients with lung cancer results in increased lung inflation. Earlier studies also reported a reduction in tumor motion with high levels of pressure [10], [11] However, we could not detect a decrease in estimated tidal volume when high pressure levels were administered. Interestingly, upon examining individual values, while the overall findings were not statistically significant, it appeared that smaller estimated tidal volumes were observed during CPAP with lower pressure levels compared to higher pressure levels. These unexpected findings might be explained by the discomfort experienced by patients when exposed to higher pressure levels, which may have hindered the acceptance of CPAP and prevented relaxation during the therapy.

No significant effects of BiPAP on estimated tidal volume was found in the overall analysis. However, when considering individual values, estimated tidal volumes reduced in 50 % of the patients compared to baseline when BiPAP with a high BURR was applied. Previous studies investigating the effects of non-invasive ventilation on diaphragm motion in healthy subjects have shown mixed results. West et al. [16] and Van Kesteren et al. [17] reported a reduction in diaphragm motion based on MRI scans, whereas Van Ooteghem et al. [14] did not find such a reduction compared to spontaneous breathing. A follow-up study from Van Ooteghem et al. [15] is the only one that investigated the effects on tumor motion in patients with lung and liver cancer. They did not report a reduction in tumor motion compared to spontaneous breathing. None of these studies provided specific information regarding the applied pressures or volumes, complicating direct comparison with our findings. Altogether, the effects of BiPAP on tidal volume may be uncertain, although it seems that BiPAP might reduce tidal volumes in certain patients.

No studies have been performed that investigated the effects of CPAP on breathing pattern variability. Given that CPAP allows the patient to completely determine their own breathing pattern, it is hypothesized that CPAP does not influence breathing variability, as confirmed by our findings. The application of BiPAP with a higher BURR is expected to decrease the variability of the breathing pattern, as supported by earlier research [12], [13], [14], [15], [16], [17]. In contrast, we did not find a significant effect of BiPAP with a higher BURR on the variability of both the estimated tidal volume and frequency. Several factors may explain these differences. First, we applied BiPAP with a higher BURR as one of the five settings applied, which may have affected the training time for each patient and therefore the patient’s ability to become accustomed to BiPAP. Second, the median percentage of patient triggered breaths was 42 %, indicating difficulty in fully controlling the patient’s breathing.

The study participants were categorized into two groups: patients with esophageal cancer or malignant lymphoma and patients with lung cancer. Patients with esophageal cancer and particularly those with malignant lymphoma are more likely to have preserved lung function compared to patients with lung cancer, as COPD is a common comorbidity in lung cancer patients [26]. We therefore deliberately included both groups to investigate CPAP/BiPAP in both contexts. The breathing frequency variability at baseline was significantly different between patient groups, while this was not the case during CPAP/BiPAP. Moreover, although not statistically significant, the median values of estimated tidal volumes and tidal volume variability were consistently higher in patients with lymphoma or esophageal cancer compared to those with lung cancer. These outcomes suggest a potentially different effect of CPAP/BiPAP in these specific groups of patients, but further research is necessary to determine which patient group would benefit the most from CPAP or BiPAP during radiotherapy.

The aim of this study was to determine the better CPAP or BiPAP setting among the applied configurations that is expected to minimize radiation exposure to healthy tissue based on its effect on estimated end-expiratory lung volume, tidal volume and variability. No configuration of the applied CPAP/BiPAP settings led to a significant effect on these factors. Therefore, more evidence should be performed to investigate which CPAP/BiPAP setting may be most beneficial during radiotherapy. Considering applied configurations in this study, BiPAP with a higher BURR emerged as the better choice for now. This setting was feasible in all patients, was well tolerated, induced a certain degree of estimated end-expiratory lung volume, and reduced estimated tidal volumes in 50 % of the patients. Although it did not lead to a reduction in breathing pattern variability, previous research suggests that BiPAP with a higher BURR has the potential to achieve this outcome. Possibly, the imposed breathing frequency should be increased more than only 3.5 breath/min higher than of spontaneous breathing, given the high percentage of triggered breaths. Furthermore, an elaborate training session should be implemented for each patient to achieve full control over the patient’s breathing with the ventilator. Our findings show that a reduction in variability may be achieved when the patient triggered breaths is significantly reduced. We are currently performing a follow-up study to investigate the impact of BiPAP with a higher BURR on tumor motion by means of 4D-CT scans. Moreover, radiation exposure to both the target and surrounding healthy tissues are evaluated.

This study has several limitations. First, this study was performed in a limited number of patients. Second, the application of the different CPAP/BiPAP settings in a fixed order, although aimed at maximizing patient comfort by gradually increasing pressure levels, could potentially impact the results. To minimize this influence, a 5-minute period of spontaneous breathing was implemented between each setting. Third, incorporating BiPAP into clinical use during radiotherapy may present challenges. To optimize the effective use of BiPAP, it is essential to provide a training session for both medical personnel involved and the patient before the initial treatment sessions. Last, the use of EIT in this study may have its limitations. EIT estimates lung volumes in a specific transversal plane located at the 4th or 5th intercostal space. The effect of CPAP and BiPAP may differ in other lung regions. It is known that tumors near the diaphragm exhibit greater motion [4]. This means that CPAP/BiPAP possibly affects the lower lobes more than the upper lobes, as confirmed by Jacobsen et al. [11]. This effect might have underestimated our findings. Furthermore, EIT remains an estimate of lung volumes and is considered a surrogate for tumor motion in this study. While earlier research showed a linear relation between tidal volumes estimated by EIT and tidal volumes measured by spirometry [18], tidal volume itself is probably not directly translatable to tumor motion, particularly in patients with lymphoma or esophageal cancer. However, it should be noted that this research was only intended as a pilot study providing valuable evidence to guide further research.

In conclusion, short-term utilization of CPAP and BiPAP in patients undergoing radiotherapy for intra-thoracic tumors is feasible and has potential for mitigating tumor motion.

## Declaration of Generative AI and AI-assisted technologies in the writing process

During the preparation of this work the authors used ChatGPT in order to improve the overall readability of the manuscript. After using this tool, the authors reviewed and edited the content as needed and take full responsibility for the content of the publication.

## CRediT authorship contribution statement

**J. Elshof:** Methodology, Investigation, Data curation, Formal analysis, Writing – original draft, Writing – review & editing. **C.M. Steenstra:** Investigation, Writing – review & editing. **A.G.H. Niezink:** Investigation, Writing – review & editing. **P.J. Wijkstra:** Methodology, Writing – review & editing. **R. Wijsman:** Conceptualization, Methodology, Writing – review & editing. **M.L. Duiverman:** Conceptualization, Methodology, Supervision, Writing – review & editing.

## Declaration of Competing Interest

The authors declare the following financial interests/personal relationships which may be considered as potential competing interests: J. Elshof reports a grant from Fisher&Paykel and grants and personal fees from Vivisol B.V., outside the submitted work. C.M. Steenstra reports no declarations of interest. A.G.H. Niezink and R. Wijsman report that the department of radiation oncology of the UMCG has research collaborations with Elekta, IBA, RaySearch, Siemens and Mirada. P.J. Wijkstra reports a consulting fee from Philips and his role as treasurer for the European Respiratory Society, outside the submitted work. M.L. Duiverman reports grants form Philips B.V., Fisher & Paykel, Vivisol B.V., Resmed Ltd and Löwenstein B.V, personal fees from Vivisol B.V., Resmed Ltd., Novartis, Chiesi, Breas, AstraZeneca, and her role as chair of Assembly 2 for the European Respiratory Society, outside the submitted work.
